# Zebra mussel beds: an effective feeding ground for Ponto-Caspian gobies or suitable shelter for their prey?

**DOI:** 10.7717/peerj.2672

**Published:** 2016-11-15

**Authors:** Jarosław Kobak, Małgorzata Poznańska, Łukasz Jermacz, Tomasz Kakareko, Daniel Prądzynski, Małgorzata Łodygowska, Karolina Montowska, Karolina Bącela-Spychalska

**Affiliations:** 1 Department of Invertebrate Zoology, Faculty of Biology and Environmental Protection, Nicolaus Copernicus University, Torun, Poland; 2 Department of Hydrobiology, Faculty of Biology and Environmental Protection, Nicolaus Copernicus University, Torun, Poland; 3 Department of Invertebrate Zoology and Hydrobiology, Faculty of Biology and Environmental Protection, University of Lodz, Lodz, Poland

**Keywords:** *Proterorhinus semilunaris*, *Dreissena polymorpha*, *Babka gymnotrachelus*, Invasional meltdown, Ecosystem engineers

## Abstract

Aggregations of the Ponto-Caspian invasive zebra mussel (*Dreissena polymorpha*) constitute a suitable habitat for macroinvertebrates, considerably increasing their abundance and providing effective antipredator protection. Thus, the overall effect of a mussel bed on particular predator species may vary from positive to negative, depending on both prey density increase and predator ability to prey in a structurally complex habitat. Alien Ponto-Caspian goby fish are likely to be facilitated when introduced into new areas by zebra mussels, provided that they are capable of utilizing mussel beds as habitat and feeding grounds. We ran laboratory experiments to find which prey (chironomid larvae) densities (from ca. 500 to 2,000 individuals m^−2^) in a mussel bed make it a more beneficial feeding ground for the racer goby *Babka gymnotrachelus* (RG) and western tubenose goby *Proterorhinus semilunaris* (WTG) compared to sandy and stone substrata (containing the basic prey density of 500 ind. m^−2^). Moreover, we checked how food availability affects habitat selection by fish. Mussel beds became more suitable for fish than alternative mineral substrata when food abundance was at least two times higher (1,000 vs. 500 ind. m^−2^), regardless of fish size and species. WTG was associated with mussel beds regardless of its size and prey density, whereas RG switched to this habitat when it became a better feeding ground than alternative substrata. Larger RG exhibited a stronger affinity for mussels than small individuals. WTG fed more efficiently from a mussel bed at high food abundances than RG. A literature review has shown that increasing chironomid density, which in our study was sufficient to make a mussel habitat an attractive feeding ground for the gobies, is commonly observed in mussel beds in the field. Therefore, we conclude that zebra mussels may positively affect the alien goby species and are likely to facilitate their establishment in novel areas, contributing to an invasional meltdown in the Ponto-Caspian invasive community.

## Introduction

The zebra mussel, *Dreissena polymorpha*, is a habitat-forming ecosystem engineer of Ponto-Caspian origin, invasive in Europe and North America (Karatayev, Burlakova & Padilla, 2002). At high densities (up to 24,000 or more individuals m^−2^) it exerts strong, multi-level impact on aquatic communities by filtering suspended matter and forming habitats for benthic organisms (Karatayev, Burlakova & Padilla, 2002). This bivalve can be beneficial for many invasive (Ricciardi & MacIsaac, 2000) and native (Karatayev, Burlakova & Padilla, 2002; Gergs & Rothhaupt, 2008) macroinvertebrates (Table 1) by providing them with suitable food (e.g. mussel pseudofaeces) and efficient antipredator shelters in a 3D structure of shells and byssally attached mussels (González & Burkart, 2004; Kobak, Jermacz & Płąchocki, 2014). Therefore, the abundance, biomass and richness of the bottom fauna associated with mussel colonies, including chironomids, oligochaetes, gastropods, amphipods and mayflies, is usually greater than in areas adjacent to mussel beds (Wolnomiejski, 1970; Karatayev, Burlakova & Padilla, 2002; González & Burkart, 2004; Kestrup & Ricciardi, 2009). Moreover, several invertebrate species, including amphipods (Kobak et al., 2009; Kobak et al., 2013), snails (Stewart et al., 1999), and mayflies (DeVanna et al., 2011a) actively prefer mussel colonies offering antipredator protection. Higher structural complexity of habitats usually decreases foraging efficiency of predators, including fish (Nelson & Bonsdorff, 1990; Mattila, 1992; Scharf, Manderson & Fabrizio, 2006). However, as the abundance of invertebrates increases with increasing habitat complexity, the highly structured substratum can constitute a trap rather than a refuge for invertebrate prey, as the exposure of potential prey can be greater due to the saturation of available shelters (Czarnecka, Pilotto & Pusch, 2014). Thus, the increased abundance of zoobenthos in a mussel bed may be beneficial for benthivorous fish, despite the lower accessibility of prey in mussel colonies. For instance, the yellow perch *Perca flavescens*, which does not feed on mussels, was experimentally shown to grow better in the presence of zebra mussel colonies due to the increased abundance of their macroinvertebrate prey (Thayer et al., 1997).

**Table 1:** Literature review of the impact of zebra mussels on the occurrence of chironomid larvae in the field.

Variable	Chironomid density (ind.) or biomass (mg) per m^2^			Mussel density (ind./m^2^)	Location	Reference
	No mussel site	Mussel site	Mussel site/no mussel site ratio			
**(A) Field experiments**						
Density	10,400	21,300	2.0	Druse	Lake Erie	Botts, Patterson & Schloesser (1996)
Density	2,100	4,400	2.1	5,400	Lake St. Louis	Ricciardi, Whoriskey & Rasmussen (1997)
Density	5,285	5,304	1.0	1,400	Lake Erie	Stewart, Miner & Lowe (1998a)
Density	4,461	8,905	2.0	5,100	Lake Erie	Stewart, Miner & Lowe (1998b)
Density	1,300	3,500	2.7	10,000	Lake Michigan	Kuhns & Berg (1999)
Density	137	293	2.1	1,000	Lake Michigan	Horvath, Martin & Lamberti (1999)
Biomass	210	780	3.7	1,400	Lake Erie	Stewart, Miner & Lowe (1999)
Density	2,103	6,942	3.3	8,400	Constance Lake	Mörtl & Rothhaupt (2003)
**(B) Simultaneous field surveys**						
Density	747	9,120	12.2	900	Lake Erie	Dermott et al. (1993)
Biomass	20	406	20.3	900	Lake Erie	Dermott et al. (1993)
Density	5,451	13,313	2.4	Druse	Lake Erie	Botts, Patterson & Schloesser (1996)
**(C) Field surveys in different years (before and after the zebra mussel invasion)**						
Density	280	360	1.3	20,500	Lake St. Clair	Griffiths (1993)^1^
Density	6	67	11.2	20,800	Lake Ontario	Stewart & Haynes (1994)^1^
Density	2.4	12	5.0	30,600	Lake Ontario	Stewart & Haynes (1994)^1^
Density	215	281	1.3	3,200	Lake Erie	Dermott & Kerec (1997)
Density	72	9	0.1	3,200	Lake Erie	Dermott & Kerec (1997)
Density	281^2^	1,946	6.9	3,900	Lake St. Louis	Ricciardi, Whoriskey & Rasmussen (1997)
Density	54^2^	431	8.0	1,500	Lake St. Francois	Ricciardi, Whoriskey & Rasmussen (1997)
Density	984	1,543	1.6	7,400	Lake Huron	Adlerstein et al. (2013)
Density	243	410	1.7	3,000	Lake Erie	Burlakova et al. (2014)

**Notes:**

1 After (Ricciardi, Whoriskey & Rasmussen, 1997).

2 Zebra mussels present at low density, < 200 ind./m^2^.

European waters have recently been invaded by several species of Ponto-Caspian goby fishes (Grabowska, Kotusz & Witkowski, 2010; Roche, Janač & Jurajda, 2013). Gobies are small, bottom-dwelling, benthivorous species (Kottelat & Freyhof, 2007) capable of living in a wide range of environmental conditions and competing with some native fishes of similar biology (Kakareko et al., 2013). Some of them, e.g. the racer goby (RG) (*Babka gymnotrachelus*) and western tubenose goby (WTG) (*Proterorhinus semilunaris*) are often found in mussel colonies (Ł. Jermacz & J. Kobak, 2014, personal observations). Thus, they could be potentially facilitated by the increased food abundance in a mussel bed. In the wild, chironomid larvae often constitute the most common and preferred dietary item of both RG and WTG (Kakareko, Żbikowski & Żytkowicz, 2005; Adámek, Andreji & Gallardo, 2007; Kocovsky et al., 2011; Vašek et al., 2014). They are also regarded as the most profitable food for benthivorous fish (Armitage, Pinder & Cranston, 1995) and may ensure a higher growth rate than some alternative food sources (Błońska et al., 2015). Moreover, chironomids have been often found to reach higher densities in mussel beds than in other adjacent substrata (Table 1). Thus, their increased availability would be likely to facilitate fish survival and establishment.

The aforementioned facilitation could be an element of the invasional meltdown. This is a community-level phenomenon, in which the presence of invasive species facilitates the establishment and amplifies the environmental impact of the others (Simberloff & Von Holle, 1999). It is supposed to result from the greater number and importance of positive interactions among aliens (e.g. habitat forming, providing food, shelters, displacing enemies, etc.) compared to their negative relationships (Simberloff & Von Holle, 1999; Ricciardi, 2001; Green et al., 2011). There are many field observations of synergistic interactions among terrestrial invaders (Heimpel et al., 2010; Edelist et al., 2012; Green et al., 2011). For freshwater ecosystems, most attention has been paid to the Ponto-Caspian fauna, which forms well established communities in Europe (Bij de Vaate et al., 2002) and North America (Ricciardi & MacIsaac, 2000). Theoretically, zebra mussels could contribute to an invasional meltdown in the non-indigenous community by providing suitable feeding grounds for gobies (Ricciardi, 2001). Nevertheless, the conditions and zoobenthic densities under which the facilitation by zebra mussels due to the increased prey abundance would exceed the negative effect of decreased prey accessibility are not known. Therefore, it is difficult to determine which situation (facilitation or suppression of feeding) is more commonly associated with mussel beds in the wild.

We conducted a series of laboratory experiments to determine what levels of chironomid prey abundance would make a mussel bed a better feeding ground for RG and WTG compared to other common substrata. This should help determine whether and at which conditions mussel beds may contribute to the meltdown phenomenon by providing suitable feeding habitats for alien fish. Zebra mussel colonies often occur on sandy substratum, with bivalves attached to one another (Garton, McMahon & Stoeckmann, 2013). Sand provides invertebrates with minimum antipredation protection (Kinzler & Maier, 2006; Kobak, Jermacz & Płąchocki, 2014), constituting potentially the easiest feeding area for fish. Thus, we compared goby feeding in a mussel bed with their performance on the sandy substratum that often occurs in the vicinity of mussel colonies and constitutes the closest alternative and the easiest feeding ground for the fish. We also tested goby feeding on a substratum made of stones resembling mussels inshape and size, to check for zebra mussel-specific effects on relationships between fish and their food.

We hypothesized that at equal food abundances the fish would consume less food from the zebra mussel substratum than from sand and stones due to efficient protection offered to invertebrates by a mussel bed (due to its solid structure resulting from byssal connections with the substratum and among mussels). However, we expected that with increasing abundance of invertebrates, the mussel substratum would become a better feeding ground than other substrata. It would contain more and more potential prey organisms, which would overcome the negative effect of their lower accessibility. Moreover, we assumed that the magnitude of the increase in food abundance needed for this shift would be within the range commonly observed in mussel colonies in the field (Table 1). An earlier study showed that RG preferred rocky substrata (stones and gravel) over mussel habitats when no food was present (Kakareko, 2011). Therefore, we hypothesized that the fish would avoid mussel habitats at equal food quantities but would switch to them when the abundance of potential prey in a mussel bed increases.

## Materials and Methods

### Experimental animals

Both goby species were caught in the Włocławek Reservoir (the lower River Vistula, central Poland, GPS coordinates of the locations: 52.615 N, 19.303 E and 52.550 N, 19.700 E) using submerged traps and electrofishing. After capture, they were transported in 10-l containers (transport time: ca. 1.5 h) to 100-l stock tanks located in an air-conditioned room with a constant temperature of 17 °C and 14L:10D photoperiod (incandescent light, 250 lx at the surface, measured with a luxometer L-20A, Sonopan Ltd., Białystok, Poland). Each species was kept separately in groups of 4–5 animals of similar body size. The stock tanks were equipped with standard aquarium filters, aerators and U-shaped shelters made of longitudinally cut PVC pipes. Weekly, we exchanged 20% of water volume and fed the fish daily with live or frozen chironomid larvae. The fish were used in the experiments after at least a month spent under laboratory conditions. Our preliminary observations have shown that a few days after placing captured fish in the stock tank, they do not exhibit any signs of stress, moving freely around the tank, occupying the shelters and taking food. We used RG of the mean total length (TL) of 6.2 cm (range 4.0–8.4 cm) and WTG of the mean TL of 5.5 cm (range 3.7–7.4 cm). The collection of fish and experiments were conducted under permit of the Local Ethics Committee (47/ŁB 625/2012).

We collected zebra mussels by diving from the same location as the fish and kept them in a 300-l aerated and filtered tank at 17 °C. They were utilized within one month after collection. The mussels were not fed in captivity, as they are known to survive such periods of starvation without tissue loss (Chase & McMahon, 1994). They attached to one another and to the substratum in the experiments, forming the desired 3D structure of a mussel bed. As a model prey organism, we used living chironomid larvae, which commonly occur and increase their numbers in mussel beds (Wolnomiejski, 1970; Mörtl & Rothhaupt, 2003). Living larvae of *Chironomus* spp. (mean length: 8.7 mm, range 6.6–11.5 mm, biomass: mean 4.79 mg, range 3.28–6.29 mg) were purchased as commercial aquarium fish food and identified to the genus level according to Wiederholm (1983). In the wild, RG feed largely on *Chironomus* spp. larvae (Kakareko, Żbikowski & Żytkowicz, 2005). There are no data about the detailed taxonomic composition of chironomids taken by WTG. However, WTG prefers slowly moving waters (Kottelat & Freyhof, 2007; Kocovsky et al., 2011), where *Chironomus* spp. larvae occur. *Chironomus* spp. have also been noted in *Dreissena polymorpha* colonies (Kuhns & Berg, 1999). We used the larvae in the experiments within a few days after purchasing. After that time, they quickly burrowed in the substratum, confirming that they were ready for use in the experiments.

We measured TLs of all the fish and 64 randomly selected chironomid larvae. We also estimated sizes of 50 randomly selected mussels and grains of mineral materials used as alternative substrata (sand and stones) as the means of two perpendicular axes of the ellipses circumscribed on their shapes (to make the measurements comparable with each other). We used ImageJ 1.40 g software (freeware by W. S. Rasband, U. S. National Institutes of Health, Bethesda, MD, USA: https://imagej.nih.gov/ij/) for measurements.

### General experimental setup and conditions

**Figure 1:**
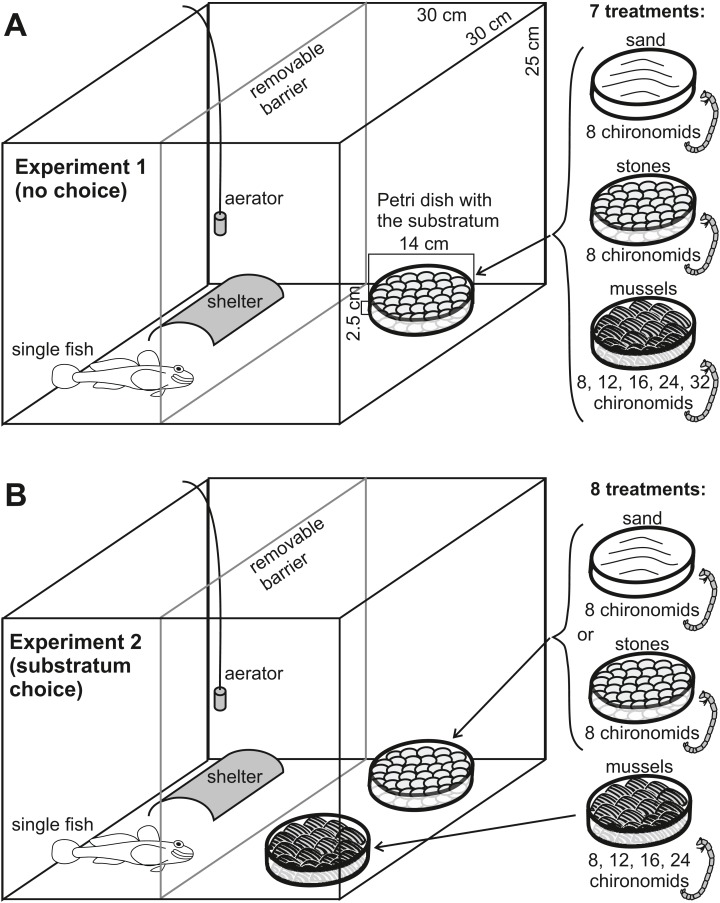
Experimental setup used in Experiment 1 (A) and in Experiment 2 (B).

We conducted the experiments in 22.5-l glass tanks filled with aerated and conditioned tap water to the level of ca. 18 cm. Each tank contained an aerator, a PVC half-pipe as a shelter and one or two (depending on the experiment) glass Petri dishes (diameter: 14 cm) acting as feeders for fish (Fig. 1). We filled the feeders with one of three types of substrata constituting feeding grounds that provided variable access of fish (predators) to chironomid larvae (prey). The following substrata were used: (1) living zebra mussels (mean size 17.9 mm, range 12.5–22.5 mm) byssally attached to one another and to the Petri dish surface; (2) stones (mean 17.3 mm, range 12.2–24.5 mm) available commercially as substratum for aquarium fish; (3) sand (mean grain diameter 0.3 mm, range 0.2–0.5 mm).

We chose stones resembling mussels with respect to their size and elongation, to check if the antipredatory protection offered by a mussel bed depended only on the size and shape of particular objects, or was enhanced by some specific traits, such as the 3D structure of byssally connected bivalves. The sand was obtained from the near-shore zone of the Włocławek Reservoir (from the site of collecting fish and mussels). We excluded any living invertebrates by flushing with water and heating for 6 h in 60 °C and removed larger particles by sieving (0.5 mm). In the reservoir, zebra mussels form druses (aggregations of individuals attached to one another) or live attached to unionid mussels on sandy substratum, thus the fish often have an opportunity to choose between these two potential feeding grounds.

We filled Petri dish feeders with the substrata up to the brim (ca. 50 mussels and stones). The use of a 2.5-cm thick substratum is justified, as it provides a considerable level of habitat complexity, with two or three layers of stones and living mussels attached to one another. Preliminary visual observations confirmed that fish readily entered the dishes and searched for food in the substratum. Thus, the presence of the dishes did not affect the foraging modes of fish.

We checked water quality parameters during the tests using a multimeter Multi340i (WTW GmbH, Weilheim, Germany). Mean water temperature (controlled by air-conditioning) was: 17.8 °C (SD 1.9 °C); conductivity: 561 μS/cm (SD 39 μS/cm); pH: 8.7 (SD 0.4); and oxygen concentration: 9.1 mg/l (SD 0.8 mg/l) or 94.7% (SD 6.4%). The light conditions and photoperiod were the same as in the stock tanks. The trials were always conducted during the light phase of the cycle between 12:00–16:00 pm.

### Types of experiments

We conducted two experiments. In Experiment 1 (Fig. 1A), we tested foraging of a single fish in the presence of a single feeder containing: (1) eight chironomid larvae in sand, (2) eight chironomid larvae among stones, (3) increasing numbers of chironomid individuals: 8, 12, 16, 24, 32 in a mussel bed (seven treatments altogether). In total, we tested 36 individuals of RG, and 39 individuals of WTG. In this experiment, we expected that the fish would feed from a mussel bed less efficiently than from mineral substrata at equal food abundances and that the mussel bed would become gradually better and better feeding ground with the increasing food quantity. We intended to determine what increase in food abundance was necessary to make a mussel bed an equal and then better feeding ground than the mineral substrata.

In Experiment 2 (Fig. 1B), we tested single fish in the presence of two feeders: one with eight chironomid larvae in a mineral substratum (either sand or stones) and the other with increasing chironomid abundances (8, 12, 16 or 24 individuals) in a mussel bed (eight treatments altogether). In total, we tested 35 RG and 39 WTG. Chironomid abundances in this experiment were chosen on the basis of the results of Experiment 1. The purpose of Experiment 2 was to determine whether fish would switch their feeding grounds to a zebra mussel bed due to increased abundance of food and at which food abundance such a change would take place. The fish behaviour was recorded using a Samsung SNB 6004 IP video camera (Samsung, South Korea).

The basic chironomid abundance used in our study (eight individuals per dish) results in the density of 520 individuals per square metre and is moderately low for this taxon (Kajak, 1997), though commonly found in the field (Johnson, Bostrom & van de Bund, 1989; Real, Rieradevall & Prat, 2000). This allowed us to test fish behaviour under conditions in which they had to search actively for their food in various habitats and were not satiated by the number of available prey items. The increasing numbers of chironomids in zebra mussel treatments were established to reflect potential changes in zoobenthos densities observed in bivalve beds in the wild (Table 1).

### Pre-experimental procedure

Two weeks before the tests we placed single fish in the experimental tanks and fed them with chironomid larvae following the same procedure as that used later in the experiment (using the same feeding dishes, times, food abundances and substrata). This allowed the fish to get used to the experimental conditions and removed the effect of learning from our results. After each feeding during this preliminary period, we checked the number of chironomids taken by fish. The fish were regarded as ready to be used in the experiments when the amount of food taken by them was stabilized in the consecutive feedings, i.e. the fish no longer increased their foraging skills due to learning. Moreover, these preliminary trials allowed estimation of the appropriate duration of the experiments.

### Experimental procedure

Before each trial, we divided the tank into two sectors with a removable glass partition (Fig. 1). Then, we put one (Experiment 1) or two (Experiment 2) dishes with the aforementioned substrata and a known number of chironomid larvae (see the subsection *Types of experiments*) into the tank, so that the fish and shelter were located in the other sector (Fig. 1). The aerator was removed during the test to avoid disturbing fish feeding and video recording in Experiment 2. After 15 min (sufficient time for chironomids to bury in the substratum, as determined by preliminary observations), we removed the partition, so that the fish gained free access to its prey. The chironomids always remained buried in the substratum and did not migrate actively in the tank. The trials in both experiments lasted for 1 h, after which we removed the substrata and counted remaining larvae.

We used a repeated measures model in which each fish individual was consecutively exposed to each experimental treatment within a particular experiment. We randomized the order of treatments among the used fish. As all the fish were accustomed to consuming chironomids from the tested substrata prior to the experiments, any differences in fish behaviour could be attributed to their responses to the treatment conditions rather than to their changing experience. Moreover, we standardized the hunger level of the fish by not feeding them for 24 h before each trial. Thus, the sequence of treatments was not likely to affect the predation success of fish in consecutive trials. This approach allowed reduction of the number of fish specimens needed for the study, to which we were obliged by the conditions of the permission from the Local Ethics Committee. Moreover, a smaller group of fish was easier to maintain in the laboratory and we could control for individual differences in feeding efficiency.

### Data analysis

After each trial, we determined the number of surviving chironomids by searching in the substratum (chironomids did not leave the feeding dishes). Substratum occupation time by fish in Experiment 2 was determined by visual examination of 60 still video frames taken from top view at minute intervals during the trial.

In Experiment 1, we tested two response variables: (1) absolute number of chironomid larvae taken by fish from substrata, to determine the food abundance at which the mussels become a better feeding ground than the mineral substrata and (2) feeding efficiency (percentage of larvae taken by fish) to assess the accessibility of food in the studied substrata. These goals were achieved by using food abundance as a categorical variable, which enabled us to find a threshold value, at which the impact of a mussel bed on fish changed. We analysed these response variables using a General Linear Model (GLM) analysis with (1) fish species as a between-subject factor, (2) fish size as a continuous variable and (3) substratum type (sand, stones or mussels with variable food abundances, seven levels altogether) as a within-subject factor (as each fish individual was exposed to each experimental treatment).

In Experiment 2, we calculated a preference index (PI) according to the formula:

$${\rm{PI}} = \left({{\rm{S}}1-{\rm{S}}2} \right)/\left({{\rm{S}}1 + {\rm{S}}2} \right)$$

where S1 and S2 are the times spent by fish in two dishes with different substrata. This index varies between −1 and 1, with 0 indicating no fish preference for any of the offered substrata. We tested two dependent variables: (1) PI of fish, to check if they changed their habitat preferences depending on food abundance and (2) absolute number of chironomid larvae taken by fish from particular substrata.

To test the PI, we used a GLM analysis with (1) fish species as a between-subject factor, (2) fish size as a continuous variable, as well as two within-subject factors: (3) mineral substratum type present in the tank (sand or stones) and (4) food abundance (four levels, 8–24 larvae in the mussel dish). Moreover, we applied sequential-Bonferroni corrected one-sample t-tests to check if the values of PI in particular treatments significantly departed from the theoretical value of 0, indicating no preference for the substrata offered.

To test the number of taken chironomid larvae, we used a GLM analysis with (1) fish species as a between-subject factor, (2) fish size as a continuous variable, as well as three within-subject factors: (3) mineral substratum type present in the tank, (4) dish (with mineral material or mussels) and (5) food abundance.

It was possible that the fish would first consume all easily accessible larvae in the mineral substratum and then switch to the mussel substratum, giving a false impression of the preference for the latter over sand with initial density of chironomids. We tested this by dividing the entire experimental period into six sections (10-min. each) and checking for the changes in fish PI with time. We used a GLM with (1) fish species as a between-subject factor, (2) fish size as a continuous variable, (3) food abundance and (4) time as within-subject variables and percentage of time spent by fish on the particular substratum as a response variable. We conducted this analysis only for the treatments with sandy substratum, as in this case the fish consumed almost all provided larvae (see the section Results). We were interested in determining if the substratum selection would depend on time. Therefore, we only considered the effects including the time factor in the model.

All percentage data were arcsine square root transformed and count data were square root transformed prior to the analyses to meet ANOVA assumptions. To control for the violation of a sphericity assumption, we applied a Greenhouse-Geisser correction to the results of the analysis if necessary (checked with a Mauchly test).

In a post-hoc procedure for Experiment 1, we intended to check differences between various substrata/food abundances as well as between fish species. If the fish size effect (continuous variable) and its interactions were non-significant, we further examined significant ANOVA effects using pairwise t-tests (for paired or unpaired data, depending on the comparison). Otherwise, we checked whether the regression slopes of the response variable on fish size for particular levels of the grouping variables significantly differed from 0. Then, (1) if both slopes did not depart from 0, we compared the group means using standard t-tests; (2) if both slopes had departed from 0, we should have checked if they were parallel, but no such case occurred in our study; (3) if only one of the slopes departed from 0, we could assume that the fish responses in both groups were different without further tests (depending on size or not). All these comparisons were sequential Bonferroni-corrected for multiple comparisons. We used IBM SPSS Statistics v. 23 for the statistical analyses.

## Results

### Experiment 1

**Figure 2:**
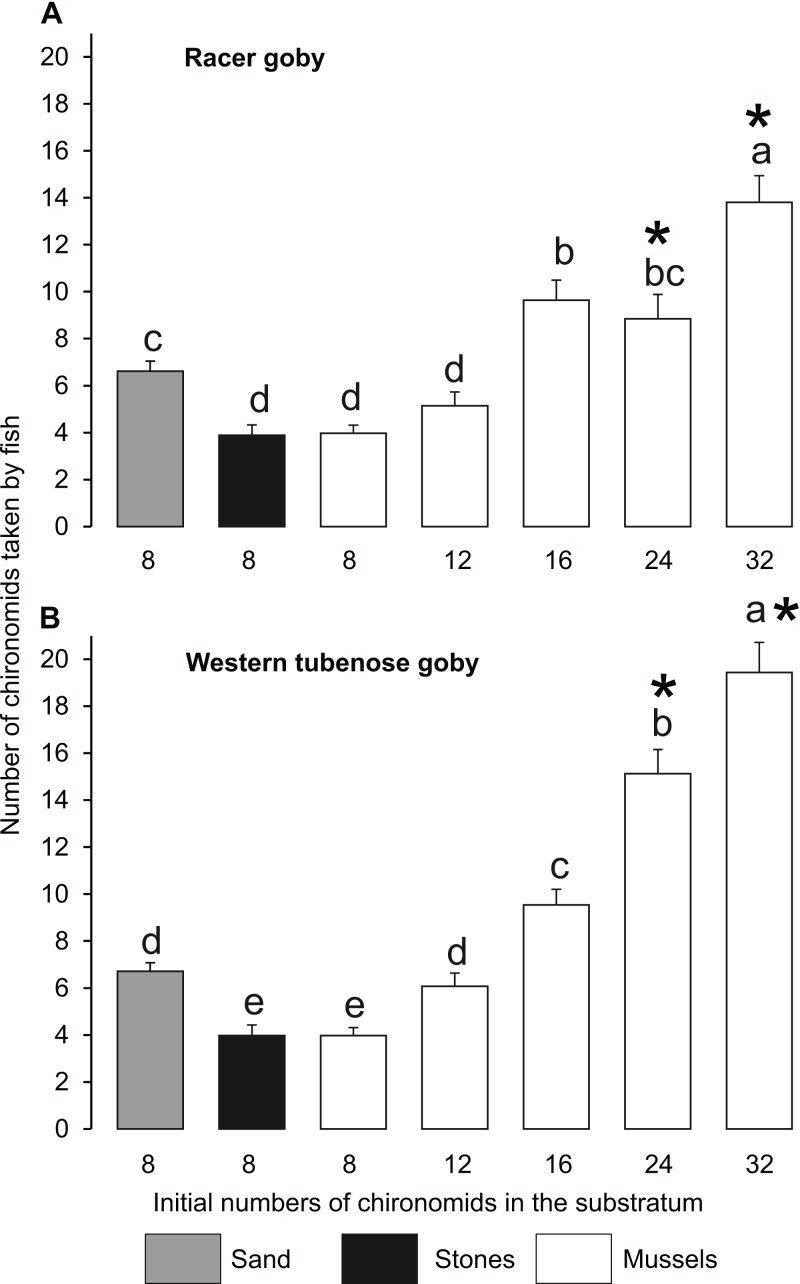
Mean (±SE) numbers of chironomid larvae consumed by the racer goby (A) and western tubenose goby (B) from different substrata in Experiment 1 (no choice experiment). Fish consumptions on the substrata labelled with the same letters (a–e) above the bars did not differ significantly from one another. Asterisks indicate treatments in which both species differed from each other.

**Figure 3:**
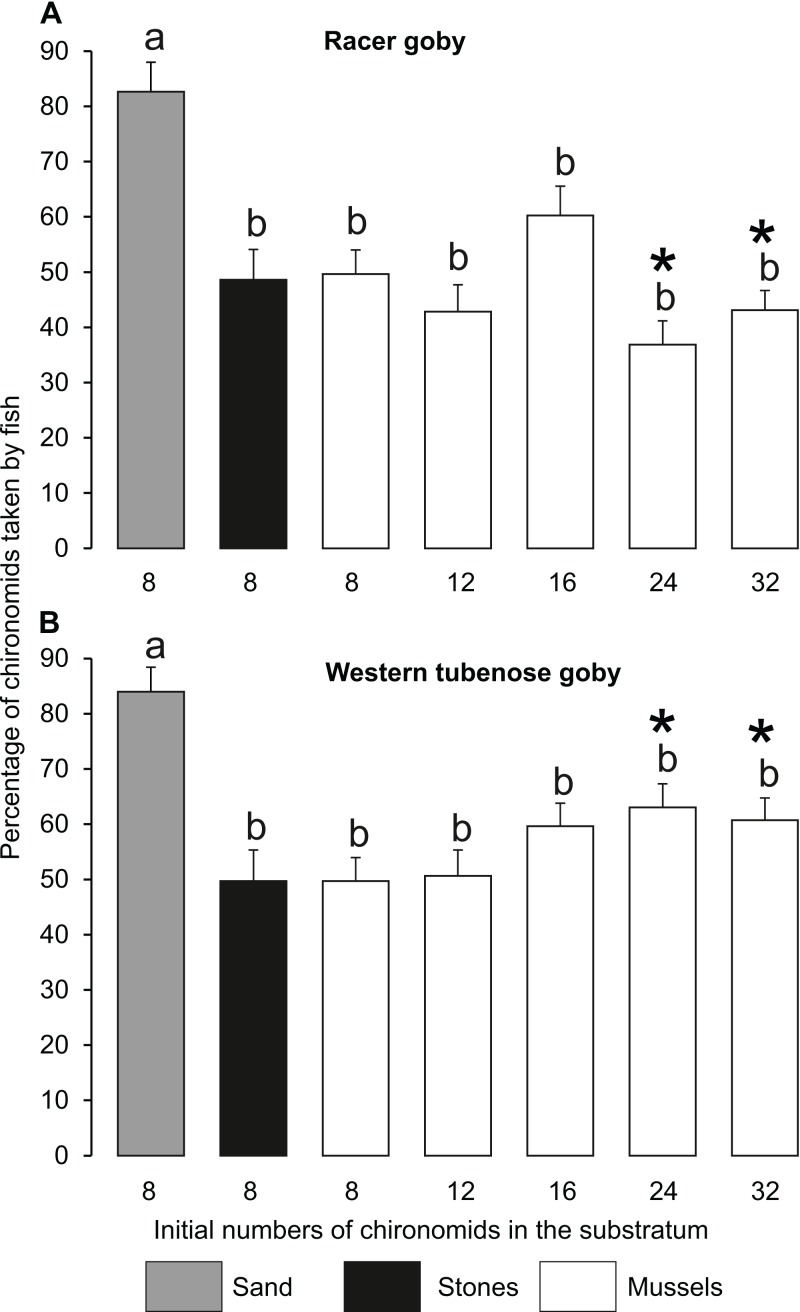
Feeding efficiency (±SE) of the racer goby (A) and western tubenose goby (B) on different substrata in Experiment 1 (no choice experiment). Feeding efficiencies on the substrata labelled with the same letters (a-b) above the bars did not differ significantly from one another. Asterisks indicate treatments in which both species differed from each other.

The number of prey individuals consumed by fish (Fig. 2) and their feeding efficiency (Fig. 3) were affected by fish species and substratum type/food abundance, as shown by significant interactions between these factors in the GLM analyses, but independent of fish size (Table 2). At the same prey abundance (eight individuals), fish consumption was highest on sand (ca. 6–7 chironomid larvae) and lower on stones and mussels (ca. four larvae). Mussels became the best feeding ground when the abundance of prey increased 2-fold (Fig. 2).

**Table 2:** General linear model analysis to test the factors affecting: (A) the number of chironomid larvae taken by fish and (B) fish feeding efficiency on various substrata in Experiment 1 (no choice experiment).

Effect^1^	df^2^	MS	*F*	*P*
**(A) Number of chironomids taken by fish**				
Sp^BS^	1	4.74	1.65	0.204
TL^Cont^	1	1.48	0.51	0.476
Error^BS^	56	2.87		
SF^WS^	6 (4.2)	1.26	1.80	0.125
SF × TL^WS^	6 (4.2)	0.18	0.26	0.911
SF × Sp^WS^	6 (4.2)	2.50	3.57	0.006*
Error (SF)^WS^	336 (237.8)	0.70		
**(B) Feeding efficiency (percentage of chironomids taken by fish)**				
Sp^BS^	1	0.31	0.71	0.404
TL^Cont^	1	0.69	1.59	0.213
Error^BS^	56	0.44		
SF^WS^	6 (4.1)	0.04	0.33	0.863
SF × TL^WS^	6 (4.1)	0.09	0.69	0.606
SF × Sp^WS^	6 (4.1)	0.33	2.60	0.035*
Error (SF)^WS^	336 (231.3)	0.13		

**Notes:**

Sp, fish species, TL, total length (continuous variable), SF, substratum type/food abundance (sand, stones or mussels with various food abundances, seven levels altogether).

1 BS, WS and Cont superscripts indicate between-subject, within-subject and continuous variables, respectively.

2 Values in parentheses are Greenhouse-Geisser corrected for sphericity (if applicable).

* Indicate significant effects.

WTG tended to utilize zebra mussel feeding grounds more efficiently than RG: it started to consume the same amount of food from mussels as from sand and more than from stones at a lower difference in food abundance between these substrata (12 larvae in a mussel bed, compared to 16 needed for RG) (Fig. 2). Moreover, WTG took greater quantities of chironomids than RG from mussel beds at the highest food abundances (24–32 larvae) (Fig. 2).

Percentage of chironomid larvae (feeding efficiency) consumed by both fish species was highest on sand (> 80%) (Fig. 3). Feeding efficiencies in the other treatments (37–60 and 49–63% for RG and WTG, respectively) did not differ significantly from one another within each fish species (Fig. 3).

### Experiment 2

#### Substratum selection

**Figure 4:**
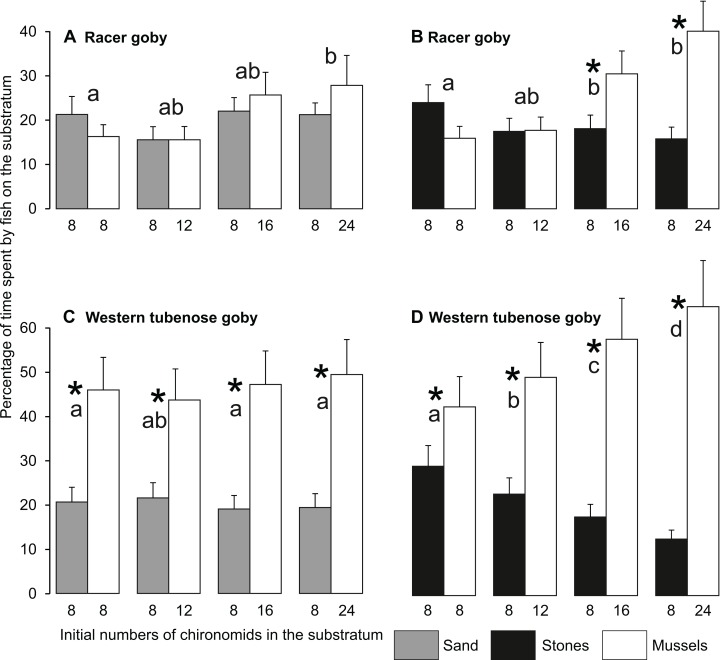
Mean (±SE) times spent by the racer goby (A, B) and western tubenose goby (C, D) on sandy and mussel substrata (A, C) as well as on stone and mussel substrata (B, D) in Experiment 2 (substratum choice experiment). Asterisks indicate significant departures of the preference index from 0 (a preference for one of the substrata offered) in particular treatments. The same letters above the bars indicate food abundances and mineral substratum types that did not differ significantly from one another with regard to the preference index.

The dish selection by fish (Fig. 4) depended on fish species, mineral substratum type (sand or stones) and food abundance, resulting in interactions between these factors in the GLM, but was independent of fish size (Table 3).

**Table 3:** General linear model analysis to test the factors affecting the percentage of time spent by fish in the two dishes present in the same tank in Experiment 2 (substratum choice experiment).

Effect^1^	df^2^	MS	*F*	*P*
Sp^BS^	1	4.16	8.11	0.006*
TL^Cont^	1	1.23	2.40	0.127
Sp × TL^BS^	1	1.11	2.16	0.147
Error (Sp)^BS^	61	0.51		
Sb^WS^	1	0.55	1.66	0.202
Sp × Sb^WS^	1	0.04	0.13	0.719
Sb × TL^WS^	1	0.22	0.67	0.417
Sp × Sb × TL^WS^	1	0.00	0.01	0.942
Error (Sb)^WS^	61	0.33		
FA^WS^	3	0.07	1.01	0.390
Sp × FA^WS^	3	0.28	4.16	0.007*
FA × TL^WS^	3	0.04	0.58	0.627
Sp × FA × TL^WS^	3	0.13	1.87	0.137
Error (FA)^WS^	183	0.07		
Sb × FA^WS^	3 (2.4)	0.50 (0.62)	5.08	0.004*
Sp × Sb × FA^WS^	3 (2.4)	0.15 (0.18)	1.51	0.224
Sb × FA × TL^WS^	3 (2.4)	0.25 (0.30)	2.50	0.085
Sp × Sb × FA × TL^WS^	3 (2.4)	0.17 (0.21)	1.74	0.178
Error (Sb × FA)^WS^	183 (148.5)	0.10 (0.12)		

**Notes:**

Sp, fish species, TL, total length (continuous variable), FA, food abundance (8–24 larvae in the mussel substratum), Sb, mineral substratum type (sand or stones).

1 BS, WS and Cont superscripts indicate between-subject, within-subject and continuous variables, respectively.

2 Values in parentheses are Greenhouse-Geisser corrected for sphericity (if applicable).

* Indicate significant effects.

RG exhibited a slight tendency to switch from the sandy substratum to mussels with increasing food quantity. No values of the PI departed significantly from 0 for the sandy substrata (Table 4), but the preference for the mussel substratum with the highest food abundance differed significantly from that observed in the treatment with the lowest food quantity (Fig. 4A). At greater food abundances (16–24 larvae in a mussel bed), RG spent significantly more time in a mussel bed than on stones (Fig. 4B), as shown by the values of the PI (Table 4).

**Table 4:** Departures of the substratum preference index from 0 (one-sample t-tests).

Substratum	Food (ind.)	Racer goby		Western tubenose goby	
		*t_25_*	*P*	*t_39_*	*P*
Sand	8	1.72	0.098	7.10	< 0.001*
	12	0.13	0.895	5.90	< 0.001*
	16	0.10	0.924	10.54	< 0.001*
	24	0.84	0.407	7.74	< 0.001*
Stones	8	0.89	0.380	3.88	< 0.001*
	12	1.26	0.219	6.95	< 0.001*
	16	3.62	0.001*	12.07	< 0.001*
	24	6.10	< 0.001*	18.87	< 0.001*

**Note:**

* Indicate significant effects (with sequential Bonferroni correction).

WTG always spent more time in a mussel bed than on both mineral substrata (Table 4). On sand, the value of the PI were irrespective of food abundance (Fig. 4C), but in the stone treatments the fish significantly increased their preference for the mussel bed with increasing food abundance (Fig. 4D).

**Figure 5:**
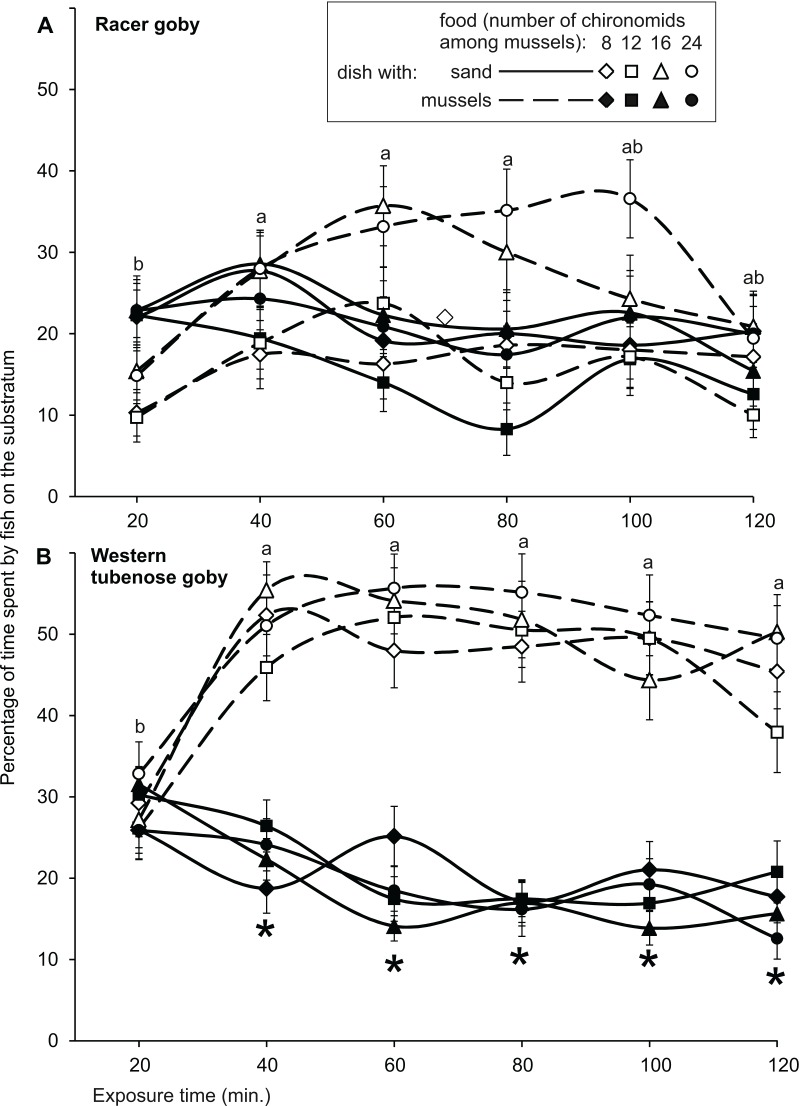
Relationship between time and occupancy of sand and mussel substrata by the racer goby (A) and western tubenose goby (B) in Experiment 2 (substratum choice experiment). Asterisks indicate significant departures of the preference index from 0 (a preference for one of the substrata offered) in particular time intervals. The same letters indicate time intervals that did not differ significantly from one another with regard to the preference index. The results of the preference index comparisons shown in the figure apply to all levels of food abundance.

Both species spent similar times on sand and in a mussel bed during the initial 10-min. period, as shown by insignificant values of the PI (Fig. 5). In the later periods, they showed a slight (RG) or strong (WTG) preference for the mussel substratum (Fig. 5), which resulted in a significant Species × Time interaction in GLM (Table 5).

**Table 5:** General linear model analysis to test the effect of exposure time and associated factors on the percentage of time spent by fish in the two dishes present in the same tank in the sandy substratum treatments of Experiment 2 (substratum choice experiment).

Effect^1^	df^2^	MS	*F*	*P*
T^WS^	5	44.09	3.93	0.002*
Sp × T^WS^	5	30.45	2.71	0.020*
T × TL^WS^	5	22.09	1.97	0.083
T × Sp × TL^WS^	5	8.41	0.75	0.588
Error (T)^WS^	305	11.23		
FA × T^WS^	15 (10.9)	8.11 (11.17)	0.78	0.698
Sp × FA × T^WS^	15 (10.9)	4.05 (5.58)	0.39	0.982
FA × T × TL^WS^	15 (10.9)	8.27 (11.39)	0.80	0.681
Sp × FA × T × TL^WS^	15 (10.9)	4.86 (6.69)	0.47	0.956
Error (FA × T)^WS^	915 (664.4)	10.36 (14.27)		

**Notes:**

T, exposure time (six intervals, 10 min. each), Sp, fish species, TL, total length of the fish (continuous variable), FA, food abundance.

Only the effects including the time factor were considered in the model to check its impact on fish behaviour.

1 BS and WS superscripts indicate between-subject and within-subject variables, respectively.

2 Values in parentheses are Greenhouse-Geisser corrected for sphericity (if applicable).

* Indicate significant effects.

#### Food consumption

**Figure 6:**
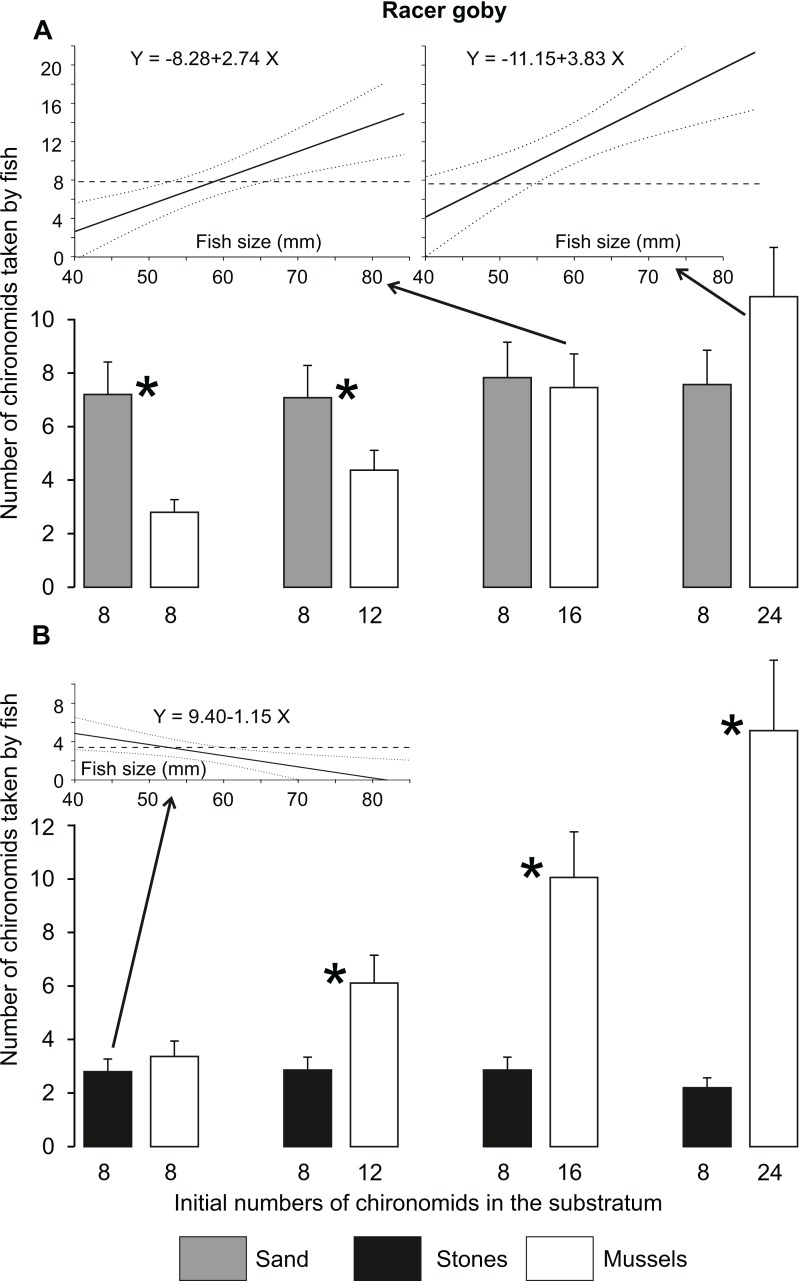
Mean (±SE) numbers of chironomid larvae consumed by the racer goby on sandy and mussel substrata (A) as well as on stone and mussel substrata (B) in Experiment 2 (substratum choice experiment). Asterisks indicate significant differences between the dishes with different substrata in the numbers of food items consumed. Inserted regression lines (with 95%-confidence intervals as dotted lines) are shown when significant relationships between the occupation time and fish size were found for particular substrata. Horizontal dashed lines in the inserted regression panels represent the mean time spent on the alternative substratum in the treatment, for which the relationship with fish size was non-significant.

**Figure 7:**
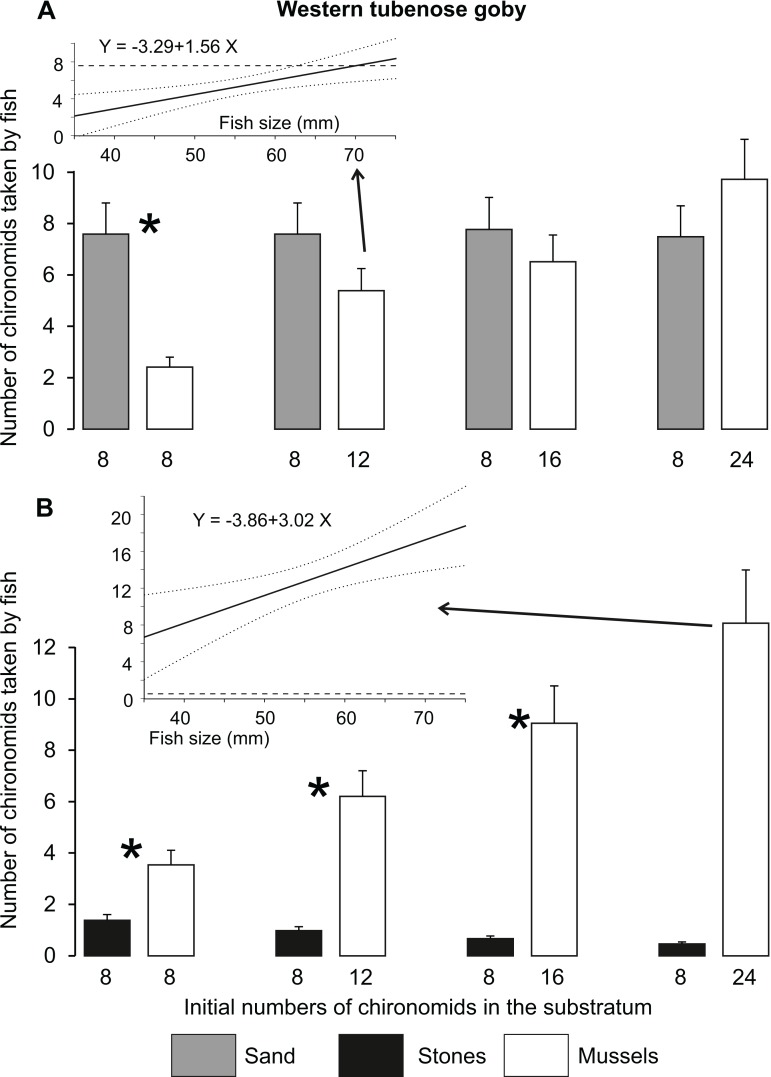
Mean (±SE) numbers of chironomid larvae consumed by the western tubenose goby on sandy and mussel substrata (A) as well as on stone and mussel substrata (B) in Experiment 2 (substratum choice experiment). Asterisks indicate significant differences between the dishes with different substrata in the numbers of food items consumed. Inserted regression lines (with 95%-confidence intervals as dotted lines) are shown when significant relationships between the occupation time and fish size were found for particular substrata. Horizontal dashed lines in the inserted regression panels represent the mean time spent on the alternative substratum in the treatment, for which the relationship with fish size was non-significant.

The fish of both species always consumed almost all larvae present in the sandy substratum (7.5 larvae on average out of eight available), whereas the number of chironomids collected from stones (1.7 larvae) was clearly lower than that observed in Experiment 1 (ca. four larvae, Figs. 2, 6 and 7). The number of chironomid larvae collected by fish from different dishes (Figs. 6 and 7) depended on fish species, fish size, mineral substratum type and food abundance, resulting in a significant interaction among all these variables in the GLM (Table 6).

**Table 6:** General linear model analysis to test the factors affecting the number of chironomid larvae taken by fish from the two dishes present in the tank in Experiment 2 (substratum choice experiment).

Effect^1^	df	MS	*F*	*P*
D^WS^	1	7.64	3.88	0.053
D × Sp^WS^	1	1.49	0.76	0.387
D × TL^WS^	1	19.74	10.03	0.002*
D × Sp × TL^WS^	1	0.57	0.29	0.591
Error (D)^WS^	70	1.97		
D × Sb^WS^	1	20.66	25.51	< 0.001*
D × Sb × Sp^WS^	1	0.24	0.30	0.587
D × Sb × TL^WS^	1	1.64	2.02	0.160
D × Sb × Sp × TL^WS^	1	< 0.01	< 0.01	0.999
Error (D × Sb)^WS^	70	0.81		
D × FA^WS^	3	0.63	1.75	0.157
D × FA × Sp^WS^	3	0.46	1.30	0.276
D × FA × TL^WS^	3	0.66	1.84	0.141
D × FA × Sp × TL^WS^	3	0.47	1.32	0.270
Error (D × FA)^WS^	210	0.36		
D × FA × Sb^WS^	3	1.66	4.54	0.004*
D × FA × Sb × Sp^WS^	3	1.38	3.80	0.011*
D × FA × Sb × TL^WS^	3	1.04	2.86	0.038*
D × FA × Sb × Sp × TL^WS^	3	1.29	3.54	0.016*
Error (D × FA × Sb)^WS^	210	0.36		

**Notes:**

Sp, fish species, TL, total length (continuous variable), FA, food abundance (8–24 larvae in the mussel substratum), Sb, mineral substratum type (sand or stones), D, feeder dish (with mussels or mineral substratum).

A difference in fish feeding from both substrata would be indicated by a significant effect of dish or its interactions, thus only the effects including this factor were considered in the model.

1 BS and WS superscripts indicate between-subject and within-subject factors, respectively

* Indicate significant effects.

RG of all sizes collected more food from sandy substratum than from a mussel bed at low food abundances (8–12 larvae in a mussel bed) (Fig. 6A). At greater food abundances (16–24 larvae in a mussel bed), the consumption of chironomids from a mussel bed increased with increasing fish size (Fig. 6A). The consumption from a mussel bed was always greater than from the stone substratum except for the smallest fish tested at the lowest food abundance (Fig. 6B).

WTG consumed more food from sand than from a mussel bed at the lower food abundances except the largest individuals with 12 larvae in a mussel bed (Fig. 7A). At greater food abundances (16–24 larvae in a mussel bed), the fish consumed similar amounts of food from both substrata independent of their size (Fig. 7A). WTG always collected more chironomid larvae from a mussel bed than from stone substratum, irrespective of food abundance (Fig. 7B).

## Discussion

### Fish feeding

In accordance with our hypothesis, zebra mussel beds turned out to be better feeding grounds than the alternative habitats when food abundance among mussels was doubled (Fig. 2). It should be noted that the number of consumed prey items increased continuously with their increasing abundance (Figs. 2, 6 and 7). This shows that the larvae remaining after the trials at the lower food abundances stayed alive because they had found suitable protection, rather than had been skipped by satiated predators.

The literature review (Table 1) shows that the abundance of chironomids in the presence of a zebra mussel bed increases in the vast majority of cases. In 70% of cases presented in Table 1, the magnitude of this increase was equal to or greater than the values found to facilitate fish feeding in our study. Similar increases in the presence of mussels have been noted for other zoobenthic taxa constituting potential food sources for gobies, such as amphipods, isopods, small snails and oligochaetes (Karatayev, Burlakova & Padilla, 1997). The presence of mussels often shifts the composition of a zoobenthic community towards larger species, which results in even greater increases in overall benthic biomass. According to Karatayev, Burlakova & Padilla (1997), an 8-fold increase in zoobenthic biomass in zebra mussel druses occurred despite a 1.5-fold reduction in the total benthos density in Lukomskoe Lake (Belarus).

At the same food abundance, goby feeding was most efficient on sandy substratum, providing prey organisms with weak protection (Kinzler & Maier, 2006; Kobak, Jermacz & Płąchocki, 2014). Feeding efficiencies of fish on the mussel and stone substrata were similar to each other (Fig. 3). This is surprising, as a mussel bed is regarded as a good antipredator shelter due to byssal connections turning it into a solid structure, which is more difficult for vertebrate predators to penetrate. Its superiority over other substrata has been shown for other prey species, such as amphipods (Kobak, Jermacz & Płąchocki, 2014). Perhaps, active amphipods, capable of clinging to solid and/or complex objects with their appendages, can utilize zebra mussel beds more efficiently to protect themselves from fish attacks, compared to less motile chironomids. Similarly, Czarnecka, Pilotto & Pusch (2014) found that relative dominance of chironomids over amphipods (*Dikerogammarus villosus*) in the diet of the perch *Perca fluviatilis* was higher on more complex surfaces. Moreover, some snails and mayflies are known to seek refuge in zebra mussel beds, preferring them over alternative substrata in the presence of predators (Stewart et al., 1999; DeVanna et al., 2011a). This also suggests the high quality of mussel beds as antipredator shelters. However, we did not show any specific protective effects of mussel colonies on chironomid prey, indicating that bivalves acted only as solid objects offering shelters against fish predation, just like stones of similar size. Thus, it seems that not all species can equally utilize the protection offered by mussel colonies. According to our study, chironomids, constituting an important component of fish diet (Armitage, Pinder & Cranston, 1995) and being facilitated by mussel colonies (see Table 1) can be relatively easily taken by fish from the mussel substratum, which suggests the potential facilitation of benthivorous fish by bivalve beds.

### Fish substratum selection

Our hypothesis predicting the change in the goby substratum preference with the increasing abundance of food in a mussel bed was partly confirmed for RG, but not for WTG (Fig. 4). It should be noted that the time spent by the fish on sand never exceeded that spent in a mussel bed (Fig. 5). Thus, they exhibited a real preference for a mussel bed, rather than switched to the mussel substratum after exhausting all available food from sand.

Contrary to our hypothesis RG did not avoid the mussel substratum even at low food abundances (Figs. 4A and 4B). This result is different from that obtained by Kakareko (2011), who tested the substratum preferences of RG (of size corresponding to the larger fish from our study) without food and demonstrated avoidance of zebra mussels in favour of other habitats (stones, gravel, sand and fine sediments). Perhaps, the addition of food, even at equal abundances in both habitats, alters the fish preferences. The substratum shift occurred when the fish were capable of consuming more food from mussels than from the alternative substratum (Figs. 4A, 4B and 6).

Compared to RG, WTG turned out to be much more strongly associated with mussel beds (Figs. 4C and 4D). Perhaps, they can perceive a mussel habitat not only as a feeding ground, but also as a shelter. Zebra mussels may constitute suitable antipredator shelters not only for invertebrates, but also for small fish if they can dig into a 3D structure of a mussel bed. For predators hunting from the water column it may be difficult to locate such a hidden prey and remove it from among the mussels connected to one another and to the substratum with byssal threads. Admittedly, we did not use piscivore signals in our study, but other cues, such as illumination of the experimental arena, could make the fish select the more protective substratum. Our results show that this might be particularly the case of WTG. The species favours areas of high structural complexity, providing numerous places to hide and is usually associated with dense vegetation or stony/rocky substrata (Prášek & Jurajda, 2005; Von Landwüst, 2006; Kottelat & Freyhof, 2007). The body of WTG (its head depth/width ratio being ca. 1.0) (Pinchuk et al., 2003a) is more laterally compressed compared to RG (head depth/width ratio of ca. 0.8) (Pinchuk et al., 2003b), which may allow it to dig in and cling into the 3D structure of a mussel bed more efficiently. Moreover, compared to RG, WTG fed more efficiently and consumed more food from the zebra mussel substratum containing large quantities of chironomid larvae, which is a common situation in the wild (Table 1). Thus, this species appears to be particularly well adapted to utilize zebra mussel colonies and is likely to benefit from their presence in its newly invaded areas, both as the feeding ground and suitable shelter.

### Relevance for the invasional meltdown phenomenon

The overall effect of the presence of zebra mussel beds on the occurrence of the Ponto-Caspian gobies seems positive: benefits from the increased prey abundance in such locations clearly exceed difficulties associated with the lower accessibility of food. Moreover, the fish (particularly WTG) exhibited active preferences for mussel beds, indicating their capability of the efficient usage of this habitat type. Thus, zebra mussels are likely to facilitate the establishment of goby species in new areas, contributing to the invasional meltdown phenomenon in the Ponto-Caspian invasive community.

Invasional meltdown is a community-level phenomenon (Simberloff & Von Holle, 1999), so a simple interaction between a few species cannot be considered as crucial evidence in this regard. However, such relationships as that described in our study do constitute the basis of the meltdown phenomenon and that is why our results may contribute to its recognition. Many interactions within the Ponto-Caspian community have often been quoted in the context of the invasional meltdown (Ricciardi, 2001). However, each relationship should be carefully checked using experimental methods to confirm its positive character and avoid spurious correlations based on purely observational studies. For instance, a recent study by Błońska et al. (2015) has shown that Ponto-Caspian gobies, commonly regarded as facilitated in their novel areas by the presence of Ponto-Caspian gammarids providing them with suitable food (Brandner et al., 2013), in fact avoid this type of prey and decrease their growth rates on such diet. Thus, this is important that our study shows experimentally that zebra mussels do affect positively alien fish species by providing them with rich feeding grounds and preferred habitats.

Obviously, native benthivores can also benefit from the increased food abundance in mussel beds (Thayer et al., 1997) and if the power of such positive interactions is similar for native and invasive species, it cannot be interpreted as the invasional meltdown (see DeVanna et al., 2011b). Nevertheless, such facilitation seems particularly important for new species, which still need to adapt to the local conditions. The possibility of utilizing habitats formed by a familar ecosystem engineer species, coming from the same region, is likely to contribute to their invasional success.

It is also known that mussel beds are utilized as antipredator shelters by Ponto-Caspian gammarids more efficiently than by local gammarid species (Kobak, Jermacz & Płąchocki, 2014), which constitutes another positive link among the zebra mussel and other invasive Ponto-Caspian species. Thus, if the invasional meltdown does take place within this community, it is mainly based on the zebra mussel and its interactions with other community members. Nevertheless, final confirmation of this phenomenon would need further studies on the relationships among other species and assessment of their relative strengths.

## Supplemental Information

10.7717/peerj.2672/supp-1 Supplemental Information 1 Datasets obtained in Experiment 1 and 2 during the study.

## Additional Information and Declarations

### Data Deposition

The following information was supplied regarding data availability: The raw data has been supplied as Supplemental Dataset Files.
